# Automatic Segmentation of Pelvic Cancers Using Deep Learning: State-of-the-Art Approaches and Challenges

**DOI:** 10.3390/diagnostics11111964

**Published:** 2021-10-22

**Authors:** Reza Kalantar, Gigin Lin, Jessica M. Winfield, Christina Messiou, Susan Lalondrelle, Matthew D. Blackledge, Dow-Mu Koh

**Affiliations:** 1 Division of Radiotherapy and Imaging, The Institute of Cancer Research, London SM2 5NG, UK; reza.kalantar@icr.ac.uk (R.K.); jessica.winfield@icr.ac.uk (J.M.W.); christina.messiou@icr.ac.uk (C.M.); susan.lalondrelle@rmh.nhs.uk (S.L.); mu.koh@icr.ac.uk (D.-M.K.); 2 Department of Medical Imaging and Intervention, Chang Gung Memorial Hospital at Linkou and Chang Gung University, 5 Fuhsing St., Guishan, Taoyuan 333, Taiwan; giginlin@cgmh.org.tw; 3 Department of Radiology, The Royal Marsden Hospital, London SW3 6JJ, UK

**Keywords:** deep learning, pelvic cancer segmentation, radiology, radiation oncology, radiotherapy planning

## Abstract

The recent rise of deep learning (DL) and its promising capabilities in capturing non-explicit detail from large datasets have attracted substantial research attention in the field of medical image processing. DL provides grounds for technological development of computer-aided diagnosis and segmentation in radiology and radiation oncology. Amongst the anatomical locations where recent auto-segmentation algorithms have been employed, the pelvis remains one of the most challenging due to large intra- and inter-patient soft-tissue variabilities. This review provides a comprehensive, non-systematic and clinically-oriented overview of 74 DL-based segmentation studies, published between January 2016 and December 2020, for bladder, prostate, cervical and rectal cancers on computed tomography (CT) and magnetic resonance imaging (MRI), highlighting the key findings, challenges and limitations.

## 1. Introduction

Owning to the recent rise of high-resolution imaging modalities such as X-ray computed tomography (CT) and magnetic resonance imaging (MRI), medical practitioners rely on spatial visualization of internal organs to evaluate disease and make timely clinical decisions. Even though radiological assessment of imaging studies is still largely visual and based on domain knowledge and expertise, there is an increasing shift towards quantitative and volumetric disease assessment for precision medicine [1,2]. This step requires accurate tissue segmentation, which can improve disease characterization through detection and division of abnormalities on images into semantically, biologically and/or clinically meaningful regions based on quantitative imaging measurements.

MRI is increasingly used for the diagnosis, staging and treatment response evaluations of pelvic cancers. With advancing imaging technologies and computer processing hardware, imaging diagnostics for cancer disease characterization, treatment assessment and patient follow-up are evolving. Quantitative imaging techniques are showing promise in providing information that can enhance the understanding of diseases and support patient care. For instance, multi-parametric MRI that combines one or more functional MR sequences is now widely used for pelvic tumors. Recently, diffusion-weighted (DW) MRI has become widely regarded as a reliable quantitative imaging technique that can provide more sensitive disease detection for the early assessment of treatment response [3]. Additionally, magnetic resonance fingerprinting (MRF) [4] has encouraged developments towards simultaneous assessment of quantitative tissue MR relaxivity.

In radiation oncology, the segmentation of organs-at-risk (OARs) and target volumes are necessary steps to aid the planning of optimal dose delivery to tumors while avoiding delivering toxicity to surrounding healthy tissues. Accurate segmentation of these structures is also vital during radiotherapy (RT) for effective image-guided treatment.

Radiomics, an image analysis approach, aims to provide additional insight from scan images that may not be fully appreciated by the human eye. It has shown potential in detecting distinct imaging phenotypes as indicators for biological behavior, therapeutic responses and treatment outcomes [5]. However, radiomics is also often reliant on disease segmentation to inform disease stratification or treatment outcomes. These applications demand increasing levels of manual region of interest (ROI) delineations which may also be subject to inter- and/or intra-operator variabilities [6], thus driving the rapid development of computer-assisted segmentation technologies to improve consistency.

Traditionally, segmentation is performed manually by radiologists and radiation oncologists, which is time-consuming [7] and it may be associated with inter- and/or intra-operator variabilities [6,8]. In RT, the time required for manual segmentation (MS) is also a rate-limiting step for adaptive radiotherapy (ART). ART is a treatment procedure that aims to account for temporal changes in patient anatomy and, potentially, tumor biology between each therapy fraction [9]. Furthermore, in RT clinics with limited resources and patient capacity, significant delays caused by MS were reported to adversely affect patient admissions as well as overall survival rates [10,11]. Therefore, significant research attention has been directed towards addressing these shortcomings in medical image segmentation.

With remarkable advancements in computer hardware, deep learning (DL) techniques have emerged as potential revolutionary solutions for clinical applications. This is due to their capabilities in learning intricate features from very large medical datasets. Adoption of advanced DL techniques by clinics may lead to significant improvements to current radiological and RT workflows. Computer-assisted segmentation technologies are continuously evolving, providing the necessity for a comprehensive review of the state-of-the-art approaches developed for cancer diagnosis, treatment planning and response monitoring. Although previous publications have provided technical reviews of recent automatic medical image segmentation approaches, [12,13,14,15,16,17] some with a particular focus on radiology [18] and radiation oncology [19,20], few studies have surveyed the clinical value and potential of DL-based segmentation approaches for different types of cancer in the pelvis. In this review, our multidisciplinary team provides an up-to-date overview of the current DL techniques used for pelvic cancer segmentation, pinpoints key achievements and discusses limitations for potential adoption in clinical practice.

## 2. Background

### 2.1. What Is Deep Learning?

Artificial intelligence (AI) is the concept and theory behind creating the ability for machines to learn and accomplish human-like intelligence [21]. DL is a sub-category of AI, inspired by the human cognition system. Unlike traditional machine learning (ML) approaches that rely on pre-programmed sets of instructions and manually-curated input data, DL offers the possibility of automatic feature extraction and learning from “raw data”. Whilst many people perceive DL to be a 21st century invention, the first wave of research on how human/animal brains learn, also known as cybernetics, started in the 1940s [22,23]. It was not until 1958 that the first fundamental component of artificial neural networks (ANNs), the perceptron, was developed, and a single-layer architecture was trained [24]. However, after a period of stagnation, the second wave of DL research, connectionism, began in the 1980s–1990s after the introduction of the backpropagation concept [25]. Backpropagation facilitated training of ANNs with one or two hidden layers for the first time. Nevertheless, due to a lack of adequate computational processing power and increased pessimism regarding real-world applications of DL in the mid-1990s, this wave of DL research was also short-lived. The current and third wave began in 2006, with development of convolutional neural networks (CNNs) [26], which allowed algorithms to be trained with significantly more efficiency than the traditional dense architectures (for example, fully-connected networks). A key innovation in this approach was the realization that sharing trained parameters (weights and biases of each perceptron) across the image through a convolution kernel enabled the development of much deeper networks for image processing than the previously available architectures [27]. Today, CNNs play a central role in AI design across a wide range of industries.

### 2.2. Deep Learning in Oncology

The interpretation of medical images is successfully undertaken by radiologists and radiation oncologists; however, their approach is often subjective and influenced by clinical experience. Depending on prior experience, humans may not be able to fully account for the range of features present on scan images. This limitation can be exacerbated by the variable appearances of tumors in cancer patients. In recent times, AI has shown potential in automatic extraction of complex image features not necessarily visible to the human eye [27].

**Figure 1:**
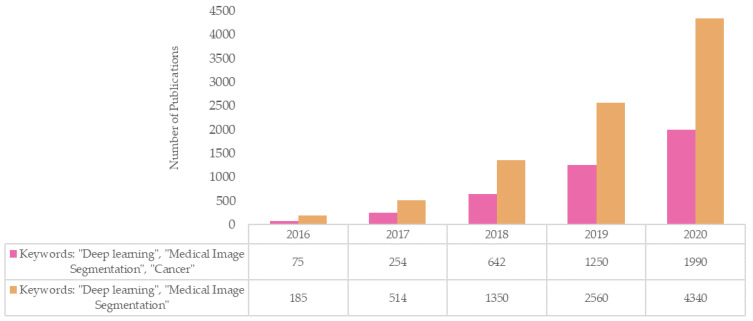
Rapid rise in the number of publications for DL-based segmentation research in medical imaging where almost half of studies were cancer-related between 2016 and 2020.

DL-based approaches have been readily deployed for clinical research since the introduction of CNNs. In oncology, the major applications of DL include tumor characterization (detection, segmentation and staging) [17,28,29,30,31,32,33], clinical outcome prediction [34,35], image synthesis [36,37] and RT dose-response modelling [38,39]. For an in-depth overview of AI applications beyond autosegmentation in radiology and radiation oncology, we refer the readers to previous studies by Boldrini et al. [19] and Meyer et al. [20]. We conducted online search with the keywords “deep learning” and “medical image segmentation” on Google Scholar for studies published between January 2016 and December 2020. The results revealed that the number of studies for DL-based segmentation research in medicine is rapidly rising. A publication search with the additional keyword “cancer” indicated that cancer research has dictated a large proportion of recent DL-based medical image segmentation studies (Figure 1).

### 2.3. Quantitative Imaging for Cancer Diagnosis, Characterization and Assessment of Treatment Response

MRI is increasingly adopted by radiologists for diagnostic and therapeutic purposes [40,41,42,43]. MRI is especially advantageous for pelvic cancer diagnosis, as its higher contrast-resolution compared with CT facilitates visualization and localization of suspicious lesions, delineation of disease extent, and subsequently enables targeted biopsy [44] and therapy planning [45]. Segmentation of target pelvic organs and tumors can be used to render disease volume, which can be further registered with patient scans from different imaging modalities for treatment planning. Tumor characterization is a broad term, which includes diagnosis, segmentation (differentiating from non-tumor tissues), staging (disease extent) and inferring its biological behavior. These applications may be enhanced by quantifying imaging characteristics such as size, shape and texture.

Tumor size measurement is important as it directs clinical decisions for the choice of treatment and evaluation of treatment response [46,47]. Disease monitoring is essential for assessing response to RT and chemotherapy treatments. The general workflow includes assessment of the tumor across longitudinal scans, and quantitative measurements according to predefined criteria (for example, the Response Evaluation Criteria in Solid Tumors (RECIST), the World Health Organization (WHO) guidelines [48]). However, unidimensional tumor measurements can be limiting, and volumetric assessment may be more robust. In addition, functional MRI techniques can be used to derive quantitative measurements that reflect on different aspects of tumor biology (for instance, DW–MRI). The apparent diffusion coefficient (ADC) is an imaging biomarker related to tissue cellularity and has been shown to be promising for early evaluation of treatment response [49,50].

Radiomic analysis of tumors, a voxel-wise assessment using imaging features derived from CT or MR images or quantitative MRI parametric maps (for example, ADC) has shown promise for evaluating tumor aggressiveness [51] and for prognostic modelling [52]. Radiomics can be used to correlate phenotypical tumor characteristics to diagnostic and/or prognostic factors. However, applications as above are reliant on the accurate segmentation of tumors, which, when undertaken manually, is both laborious and subjective [6,53]. Hence, automated and robust tumor segmentation tools are highly desirable for the rapid quantitative characterization of cancers.

### 2.4. Radiotherapy Treatment (RT) Planning and Optimization

CT remains the mainstay imaging modality for RT treatment planning due to its high acquisition speed and high spatial resolution, and provides relative electron density information. However, CT lacks the desired soft-tissue contrast for accurate delineation of organs and tumors where electron densities of neighboring structures are not significantly different. Therefore, in radiation oncology, gross tumor volumes (GTVs) are sometimes derived from MRI for more accurate delineations [54]. The examples of GTVs of MRIs and CTs are shown in [55] and [56]. Within a treatment planning system (TPS), the radiation oncologist initially identifies the target volumes and OARs. A series of target volumes are defined according to the criteria reported by the International Commission on Radiation Units and Measurements (ICRU) [57], based on initial tumor identification, expanded to include subclinical disease, and, finally, a planning target volume (PTV) to account for day-to-day setup variation. Consistent identification of these target volumes during treatment using automated segmentation frameworks could help to reduce the expansion margins currently employed, and therefore limit irradiation of normal tissue. Despite defined delineation protocols, inter-observer variation in target delineation is the greatest source of uncertainty, necessitating an additional margin of error to be employed in creating the PTV [58]. Image-guided radiation therapy (IGRT) techniques are increasingly attracting research attention to mitigate these shortcomings and allow clinicians to adapt treatment plans prior to and/or intra-fraction to objectively monitor the position of target volumes. ART is a potentially promising treatment procedure that suits tumor sites with large inter-fraction deformability (for example, bladder, cervix, prostate, rectum); it allows better sparing of the OARs from radiation toxicity. However, the need for redefinition of ROIs for each ART fraction poses a significant limitation in routine treatment workflows. Thus, fast accurate and automatic segmentation of ROIs is considered the central requirement for the adoption of ART in clinical practice.

### 2.5. Automatic Image Segmentation

Traditional segmentation algorithms were low-level image feature extractors (for example, intensity-based and edge-based). Common methods included intensity thresholding, region growing and edge-detection, which selected semantic image regions solely based on visual information from input images. More advanced mechanisms, such as uncertainty and optimization algorithms, were introduced to overcome the limitations associated with previous heuristic approaches. For instance, deformable models (for instance, active contours [59], level-set algorithms [60]) were developed to allow contours to expand/contract to include distinctive regions. Graph-based methods (for instance, graph cuts [61], watershed algorithm [62]) applied the principles of game theory for segmentations based on inter-voxel relationships. Probability-based algorithms (for example, Bayesian classifier [63,64], Gaussian mixture models, clustering, k-nearest neighbor [65], ANNs) were developed to automatically assign individual voxels to different classes. However, these approaches lacked contextual information, which led to suboptimal segmentations. Although these algorithms can be combined with Markov random field models to alleviate this drawback [66], the success of these techniques is strongly correlated with manual human interactions. Atlas-based approaches were proposed to incorporate prior knowledge in segmentation algorithms. Early atlas-based algorithms consisted of a single atlas (a manually defined set of regions on an existing reference image dataset) from which the contours from the reference image were transferred to the new image following deformable registration [67]. However, segmentation heavily relied on registration accuracy and organ morphology, leading to suboptimal contours, especially for patients with unusual anatomy.

Later approaches proposed the use of more advanced atlas selection techniques [68,69], selection of an atlas containing average patient anatomy information [70] and multi-atlas segmentation as prior knowledge [67,71]. Currently, multi-atlas algorithms are the most common techniques used in defining target tumor volumes [72]. Nonetheless, the major limitations with atlas-based methods remain the considerable computational and time constraints. Currently, an array of software programs is available for automatic registration and segmentation of tumors using pre-defined templates and deformable contour propagations [73,74]. However, these programs are not suitable for pelvic cancers due to unclear boundaries between the gross tumor and subclinical malignant regions [75]; tumor contouring heavily relies on clinicians’ experience.

**Figure 2:**
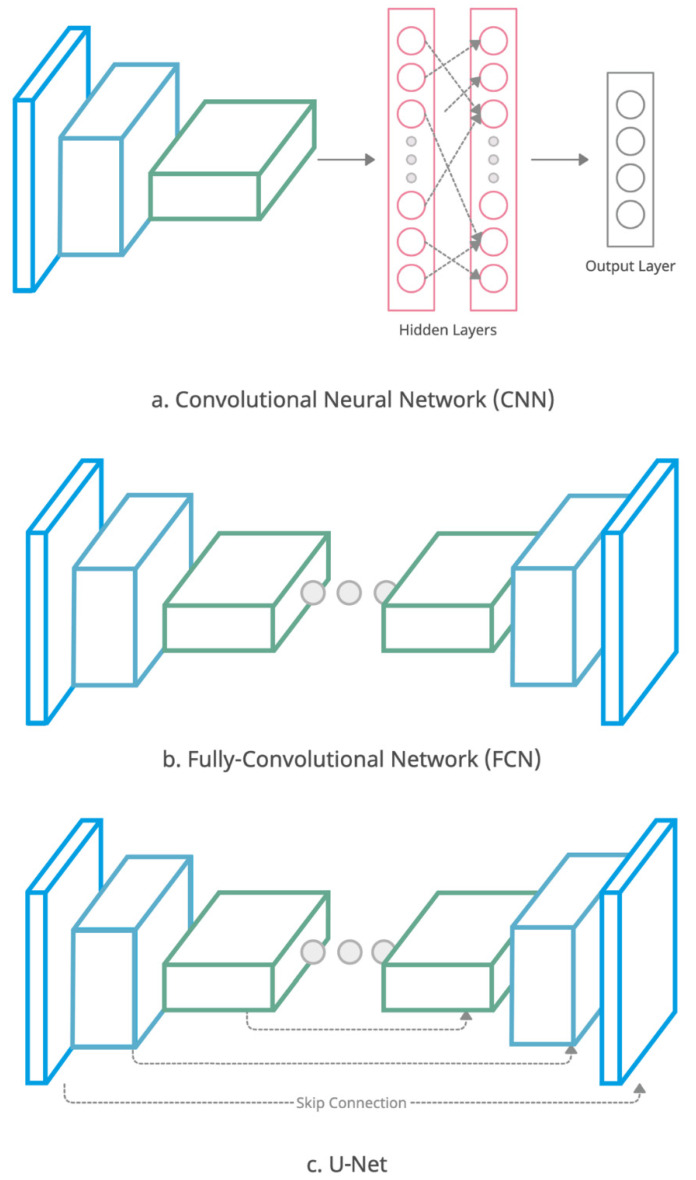
Illustration of (**a**) convolutional neural networks (CNN) with fully-connected final layers for classification tasks, (**b**) fully-convolutional network (FCN) for image-to-image or image-to-mask translations and (**c**) U-Net architecture with skip connections between encoder and decoder in the network for more efficient feature extraction/reconstruction than FCN.

DL-based segmentation methods have shown enormous potential in computer-assisted clinical applications due to their ability to learn complex information from very large datasets. Unlike traditional auto-segmentation approaches that rely on human-defined heuristics, CNNs are able to automatically capture the pertinent information contained within existing (training) datasets needed for successful segmentation. CNNs are generally formed by stacking several layers (for example, convolutional/deconvolutional, fully-connected, pooling, upsampling layers), each of which perform a key operation on the input images (See Figure 2a for a basic CNN classification architecture). Conventionally, CNNs performed pixel/voxel-wise classifications to isolate independent pixels/voxels in order to form ROIs from images. However, this was computationally inefficient due to repetitive iterations of identical convolutional operations throughout images. In 2015, Long et al. [76] introduced fully-convolutional networks (FCNs) to mitigate the limitations with fully-connected layers (final set of layers in CNN) for extracting local spatial correlations. The FCN architecture includes symmetrical encoding and decoding paths which enable learning of both low- and high-level feature representations in images (Figure 2b). One of the most popular DL architectures used for medical image segmentation is U-Net [77], which is a special type of an FCN with the addition of skip connection pathways between encoders and decoders (Figure 2c). In recent years, many variations of U-Net and FCNs have been published to enhance segmentation performance across a wide range of medical applications. Typical examples include 3D U-Net [78], V-Net [79], DeepMedic [80] and DeepLab [81]. We direct the readers to [12,14,18,82] for comprehensive technical overviews of the DL architectures used in recent medical research.

#### Evaluating the Quality and Success of Segmentation

One of the most broadly-used metrics for comparing automatically-generated contours with the ground-truth is the Dice similarity coefficient (DSC) [83]. DSC evaluates the overlap between two sets of contours (A and B) divided by their mean area. DSC ranges from 0 to 1, where higher values correspond to more accurate segmentation results (Equation (1)). It considers both false positives and false negatives; therefore, it is superior to accuracy which only incorporates correctly-identified pixels/voxels in images. Another variation of DSC reported in the literature is the surface Dice similarity coefficient (SDSC) [84] that, with the addition of parameter τ, incorporates inter-observer variabilities in measuring the overlap between two surfaces. Intersection-over-union (IoU) or Jaccard index (JI) is another segmentation metric reported in the literature [85] (Equation (2)).

(1) $$\mathrm{DSC}=\frac{2\left|A\cap B\right|\;}{\left|A\right|+\left|B\right|}$$

(2) $$\mathrm{IoU}=\frac{A\cap B\;}{A\cup B}$$

One limitation associated with volume-based segmentation evaluation metrics (for instane, DSC, IoU) is the lack of sensitivity to the boundary of contours with potential spatial co-location. This is especially important in radiation oncology, where the contours of adjacent organs/target disease volumes may signify the difference between irradiated and at-risk regions. Therefore, distance-based metrics are used as additional indicators to assess segmented contours. The Hausdorff distance (HD) [86] is defined as follows (Equations (3) and (4)):

(3) HD(A,B)= max(h(A,B),h(B,A))

(4) $$h\left(A,B\right)=\underset{b\in B}{\max}\left(\underset{a\in A}{\min||}a-b||\right)$$

where h(A,B) is the largest distance from a point in A to the nearest point in B. 

HD is generally inversely correlated with segmentation accuracy. Additionally, the mean surface distance (MSD) is Equation (5):

(5) $$\mathrm{MSD}=\frac{1}{\left|A\right|+\left|B\right|}\left(\underset{a\in A}{\sum }\underset{b\in B}{\min\;}d\left(a,b\right)+\underset{b\in B}{\sum }\underset{a\in A}{\min\;}d\left(b,a\right)\right)$$

where d(a,b) corresponds to the distance between points a and b.

In the following sections, we review DL-based segmentation publications for different cancer types within the pelvis.

## 3. Literature Review

The literature review in this study was conducted by an initial article search in PubMed/Medline and ScienceDirect databases with the keywords “deep learning”, “segmentation”, “cancer”, “organs at risk”, “radiation oncology”, “radiology” and “radiotherapy”, and a subsequent manual reference check of the relevant publications. This approach aimed to create a clinically-oriented overview of the DL-based pelvic segmentation algorithms currently used in pelvic cancers. The exclusion criteria for the retrieved publications were as follows:

- non-DL segmentation techniques;
- segmentation applied to sites other than the pelvis;
- no training/validation of methods on real patient data;
- image modalities used other than CT and MRI;
- full articles published in languages other than English;
- no clinical application focus or published outcome

Overall, we included 74 relevant studies on bladder, cervical, prostate and rectal cancer segmentation applications to present a comprehensive review of the state-of-the-art approaches.

### 3.1. Bladder Cancer

Segmentation of the inner and outer bladder wall and tumors on MRI plays an important role in the diagnosing and staging of urinary bladder cancer, as it provides excellent soft-tissue visualizations. On CT, bladder disease segmentation can provide clinicians with insight on cancer tumor progression and treatment response monitoring [87,88]. Bladder segmentation on MRI is a challenging task due to large inter-patient anatomical variations as well as imaging signal inhomogeneities in the urine caused by motion artefacts and unclear soft-tissue boundaries [89,90]. The difficulty of segmentation increases with the presence of cancer in the bladder. Previous studies performed automatic bladder segmentation using adaptive Markov random field [91], adaptive shape prior constrained level set [92] and statistical shape-based algorithms [33]. However, a lack of generalizability due to large anatomical discrepancies in patient populations and the need for manual feature and parameter selection prevented their widespread clinical adoption.

**Figure 3:**
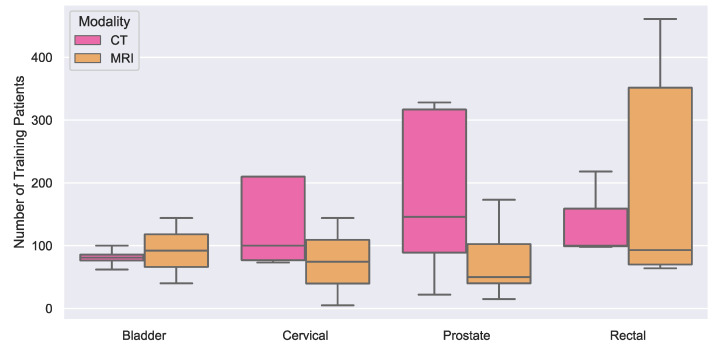
Boxplot of number of training patients used in segmentation applications for bladder (CT studies: 4, MRI studies: 2), cervical (CT:2, MRI:5), prostate (CT:12, MRI:40) and rectal (CT:2, MRI:6, CT/MRI:1) cancers. The average number of training patients was 165 from the 74 reviewed studies. The outliers were excluded from this figure for visualization purposes.

To overcome this limitation, Ma et al. [88] developed a U-Net that improved bladder segmentation on CT compared with their previous combined CNN and level-set segmentation algorithm [93], particularly in lower-resolution images and scans from patients with locally-advanced urinary bladder cancer. However, the authors reported that contrast-enhanced CT images added more complexity to segmentation due to the variable appearance of the bladder based on the effects of urine motion and filling from excreted contrast material. Xu et al. [94] proposed a 3D bladder segmentation framework on CT involving a fully-connected conditional random fields recurrent neural network (CRF–CNN) and fine-localized bladder probability maps; they reported that their approach outperformed the state-of-the-art V-Net algorithm for volumetric segmentation of the bladder. On the other hand, only the study published by Dolz et al. [95] incorporated DL for bladder cancer segmentation on MRI. The authors developed a U-Net to perform multi-region semantic bladder segmentation and reported that this approach outperformed traditional non-DL autosegmentation techniques. We hypothesize that the paucity of published studies for use of DL in bladder cancer segmentation may be due to the lack of public and annotated datasets, as well as the lower prevalence of the disease compared with other pelvic cancers (see Table 1 and Figure 3).

### 3.2. Cervical Cancer

Segmentation of cervical tumors remains a challenging task due to large geometrical variations in patient populations and indistinctive soft-tissue boundaries. Previous studies have reported the utility of DW–MRI and ADC for cervical cancer staging, histological grading and nodal status evaluations [158]. Despite growing interest in quantitative assessment of tumors in radiology, to date, only one previous study, by Lin et al. [17], incorporated the use of DL for automatic segmentation and radiomic feature extractions of cervical tumors from ADC maps. The authors demonstrated that their framework outperformed previous ML techniques by a factor of two, potentially providing clinicians with an automated tool to minimize tumor delineation (GTV equivalent) discrepancies. Moreover, Breto et al. [102] developed a Mask R–CNN framework for automatic segmentation of OARs and GTVs for MR-only RT treatment planning for patients with locally advanced cervical cancer. The authors reported that while the generated contours for the cervix, rectum, bladder, uterus, femur and sigmoid were in good agreement with expert MS, their network underperformed for segmenting smaller and less distinctive soft-tissue structures such as the vagina, parametrium and the mesorectum. However, their results were only based on five test patients and not clinically validated. The considerable segmentation complexities in cervical cancer as well as the lack of high-quality and annotated databases may have also contributed to the low numbers of studies for DL-based segmentation of cervical tumors on MRI (Table 1).

In the RT literature, Wang et al. [99] proposed a 3D U-Net model for clinical target volume (CTV), which typically encompasses the tumor, cervix, uterus, ovaries and parametria, and OAR delineations on CT from 25 patients, and suggested that their automatic contours were as accurate as MS performed by a clinical resident with 8 months’ experience. Liu et al. [97] developed a 3D U-Net architecture for segmentation of OARs and reported that over 90% of their generated contours were “highly acceptable” for RT planning through expert oncologist evaluation (>15 years of experience). However, this network underperformed for CTV delineations. In a later study, the authors developed a dual-path U-Net network (DpnUNet) consisting of more hidden layers in order to make it more suitable for CTV segmentations where tissue boundaries are unclear. However, despite promising segmentation results, their framework was only evaluated on patient scans from a single institution. In contrast, Rhee et al. [101] used a V-Net [79] model to generate CT treatment plans and reported that their algorithm achieved on average 80%, 97% and 90% clinical acceptance rates for primary CTVs, OARs and bony structures, respectively. Their framework was validated on 30 cervical cancer patients scanned across three hospitals. The list of the publications for cervical cancer segmentation studies is shown in Table 1.

### 3.3. Prostate Cancer

Previous review studies have investigated various automatic segmentation approaches. However, only one previous study, published by Almeida and Tavares [16], provided a systematic review of advances in prostate segmentation, and included 28 publications for studies until 2019 (CT: 9, MRI: 19). This study provides an up-to-date review of 52 publications on prostate and/or prostate cancer segmentation (CT: 12, MRI: 40) (see Table 1). Based on our literature search, it is apparent that in recent years, the clinical attention on segmentation of prostate cancers has gravitated towards MRI due to its unparalleled soft-tissue contrast. There remains limited literature for automatic segmentation of prostate cancers themselves, in part because of the technical challenges imposed by the relatively small size of the tumors, background changes within the prostate gland also because major treatments (for example, RT) are usually directed towards the whole prostate gland rather than the focal disease. However, as automated decision support tools for prostate cancer diagnosis in MRI are being developed, together with internal radiation boost for prostate cancer and other focal therapies becoming more widely used, prostate cancer segmentation will become increasingly important.

At present, whole prostate gland (WG), central gland (CG), transition zone (TZ) and peripheral zone (PZ) segmentations have been developed to aid disease assessment and prostate cancer staging [159]. WG segmentation is also the basis for RT planning. Earlier prostate zonal segmentation algorithms included active appearance [160], continuous max-flow [161] and C-means algorithms [162]. However, these techniques failed to generalize to patient populations from multiple institutions. Due to high clinical demand and technology advancement, DL rapidly found its way into prostate segmentation research. Amongst the MRI-based prostate segmentation studies in our review, 33 studies performed segmentation of WG. However, from these publications, only eight studies also investigated CG, TZ and PZ segmentations [115,120,121,125,126,127,134,147]. In these studies, WG segmentation accuracy was superior to PZ and TZ due to large anatomical variations and indistinguishable soft-tissue boundaries. Moreover, only four studies provided results on prostate cancer segmentation on MRI [117,125,134,145] (see Table 1).

From the 40 reviewed MRI-based prostate segmentation publications, 32 and 4 used 2D and 3D imaging data for training their DL networks, respectively, whilst one study used a combination of 2D and 3D input MRI to train their segmentation algorithms. Additionally, the MR imaging acquisition mode was unspecified for one or all MRI contrasts in three studies. Although using volumetric images for training incorporates vital spatial information for organs, it requires considerable computational resources to facilitate training. One advantage of training DL algorithms with 2D convolutional kernels is the ability to use knowledge transfer (transfer learning) from previous models trained on natural images in order to achieve greater segmentation performance. Tian et al. [29] proposed a variant of FCN called PSNet, and through transfer learning, achieved satisfactory results. Zhu et al. [144] developed a CNN with deep supervision to better capture multi-level feature maps. Attempting to investigate the performance of generative adversarial networks (GANs), Birbiri et al. [116] proposed a conditional GAN (cGAN) and reported that their algorithm with a U-Net generator outperformed the standalone U-Net model. On the other hand, benefiting from volumetric model training, Milletari et al. [79] developed a 3D CNN called V-Net to perform prostate gland segmentation. Feng et al. [137] used a multi-task FCN for training in a semi-supervised manner to overcome lack of adequate training data. Zhu et al. [118] proposed a boundary-weighted strategy to enforce feature learning at the base and apex of the prostate from a limited training dataset.

The considerable difficulty in automatic delineation of pelvic organs have inspired the introduction of various segmentation challenges. These include PROMISE12 [163], ASPS13 [164] and PROSTATEx [165]. Amongst the reviewed articles in this study, 28 publications used public datasets for network training and/or validation. For example, Yu et al. [166] developed a 3D CNN with mixed long and short residual connections that enabled high training efficiency and superior feature learning capability from small training datasets. This framework outperformed other proposed algorithms in the PROMISE12 challenge in 2018. Moreover, Brosch et al. [139] developed a framework containing regression-based boundary detection and CNN-based prediction of the distance between a surface mesh and its associated boundary point which ranked first place in the PROMISE12 challenge in 2019. Geng et al. [124] proposed an encoder-decoder architecture with dense dilated pyramidal pooling, and, after validating their technique on PROMISE12 and ASPS13 datasets, reported that their framework outperformed the then state-the-of-art algorithms for segmentation. Dai et al. [117] developed a region-based CNN (Mask R–CNN) and suggested that their approach was able to perform end-to-end segmentation of the prostate as well as the highly suspicious lesions from the PROSTATEx repository. Based on our literature research, it is evident that the introduction of segmentation challenges along with public and annotated databases for prostate cancer have encouraged research from the wider ML community. The list of available databases and publications for prostate segmentation are shown in Table 2.

Traditionally, OARs and segmentation for RT planning in prostate cancer were performed using volumetric deformable model surface [170], organ-specific modelling [171] and atlas-based techniques [74]. However, contouring through these techniques was poor for patients with abnormal anatomy and data from external institutions, hence hindering the possibility of their integration for online adaptive treatments. Therefore, recent studies have employed DL-based algorithms to develop more efficient, generalizable and consistent segmentation pipelines. The current RT planning workflow uses CT for ROI contouring and radiation dose estimations. Hence, despite poor soft-tissue contrast, segmentation on CT remains desirable. Ma et al. [31] proposed a framework combining a 2D CNN with multi-atlas label fusion to segment ROIs on CT. Balagopal et al. [112] used a 2D–3D hybrid U-Net model containing aggregated residual networks (ResNeXt) to enhance algorithm feature learning capability, and achieved an average DSC of 0.9. However, this was only based on ground-truth data defined by only one expert. Wang et al. [107] proposed a 3D FCN with boundary sensitive representations for enhanced organ-specific feature learning and verified their results based on data from 313 patients, acquired from multiple CT scanners. On the other hand, Dong et al. [106] used a Cycle Consistent Generative Adversarial Network (Cycle-GAN) to generate synthetic MRI from CT to enhance their algorithm’s soft-tissue learning capability. However, the impact of registration for contour propagations from MRI to CT was not reported. MRI-only RT planning was also proposed to mitigate these geometrical uncertainties. To the best of our knowledge, there are no public CT databases for prostate segmentation and RT planning.

### 3.4. Rectal Cancer

MRI is the technique of choice for the diagnosis and preoperative staging of rectal cancer [172]. MRI is more accurate in the diagnosis, staging and treatment planning of rectal cancer compared with CT, and also provides quantitative tumor assessment, which can inform treatment response assessment and disease outcomes [173]. Although in recent years, numerous studies were published for automatic contouring of pelvic tumors [101,174,175,176,177], only a few reported to address rectal cancer [32,152,178]. Based on our article search, nine studies incorporated DL for rectal cancer segmentation applications (CT: 2, MRI: 6, MRI/CT: 1) (Table 1). Trebeschi et al. [157] published the first CNN-based rectal tumor segmentation study on multi-parametric MRI. Their framework included classification of fixed patches and segmentation of the identified voxels. Although this approach was designed to reduce image redundancy, it ignored context information which adversely affected their network’s generalizability in cross-institution model evaluations. Huang et al. [156] developed a volumetric hybrid loss fully-convolutional network (HL-FCN) that used Dice-based loss to overcome class imbalance in their training data, however their results were not clinically evaluated. Jian et al. [28] proposed an FCN-based segmentation framework and used transfer learning to outperform the conventional U-Net architecture for rectal tumor segmentation on MRI. Similarly, Wang et al. [154] deployed an FCN model from a pre-trained ResNet50 model to enrich hierarchical feature extraction during network training. The authors evaluated their results on 107 patients from four centers and reported that their network was superior than U-Net for tumor contouring. Unfortunately, due to a shortage of public databases, direct and meaningful comparison of these algorithms for rectal cancer segmentation remains a challenging task.

To date, only three studies were published on uses of DL for rectal cancer RT treatment planning on CT images. Men et al. [152] proposed a 2D CNN with dilated convolutions and suggested that their network outperformed the traditional U-Net architecture. However, the authors reported that their model failed to accurately perform colon and intestine segmentations due to large inter-patient anatomical variabilities and inhomogeneous distribution of the contrast material and gas in these structures. Song et al. [32] investigated DeepLabV3+ and ResU-Net architectures for OARs and CTV segmentations, and suggested that while automatic contouring using these models outperformed the framework proposed by Men et al. [152], they offered different advantages for feature extraction and contouring of pelvic structures. While ResU-Net was reported to be an effective algorithm for segmenting visually distinctive structures (for example, femoral heads, bones), DeepLabV3+ achieved superior segmentation performances for soft tissues with unclear boundaries (for example, bladder/small intestine). Their results were in line with a later study by Men et al. [151], who employed cascaded convolutions along with spatial pyramid pooling (SPP) to enhance CTV delineations. However, both of these techniques were based on 2D training that disregards the inter-slice spatial information of OARs and tumor volumes for training.

## 4. Discussion

Significant research attention has recently shifted towards bridging the gap between computer vision and patient care. In this review, we presented an overview of the recent DL-based automatic segmentation algorithms used in bladder, cervical, prostate and rectal cancers from 74 studies. We included studies that incorporated in their DL-based analyses the use of input CT and/or MR images. CT is widely used as the desired imaging modality for radiation dose estimations and RT treatment planning. However, the inadequate soft-tissue contrast on CT necessitates the concurrent adoption of MRI for enhanced visualization of pelvic structures to improve the accuracy of tumor definition, leading to potential segmentation uncertainties caused by mis-registration. On the other hand, the major limitation with cancer tumor segmentation on MRI remains the difficulty in confidently identifying abnormal structures from healthy tissues. This is due to highly variable inter-patient geometrical appearance and potentially poorly-defined soft-tissue boundaries.

Unfortunately, unlike DL applications for natural images, access to medical images for training and evaluating algorithms is restricted. This limitation is largely due to patient data privacy and labor-intensive ground-truth contour definitions. Difficulty in accessing high-quality and adequately large in-house repositories may hinder research motivation from the wider ML community. We demonstrated, through comprehensive literature review, that, although partially due to higher prostate cancer prevalence, the introduction of grand MRI segmentation challenges and publicly-accessible datasets have played an important role in driving prostate cancer research forward. Regrettably, to the best of our knowledge, there are no public and annotated repositories for other pelvic cancer types (MRI or CT). Therefore, global and institutional efforts are necessary to initiate public datasets to encourage future widespread research. However, appropriate quality control and external expert auditing need to be in place to ensure data are of high quality [179,180].

Lack of common datasets also creates difficulty in fairly and accurately comparing new DL algorithms with previous research studies. Based on the reviewed articles, the MRI acquisition mode (2D or 3D) for five studies were labelled as “unspecified’ since insufficient acquisition information was provided for training MR images. Whilst DL network dimensionality and architecture selection are important for the success of automatic segmentation algorithms, the understanding of input data as well as the reproducibility of network outcome are of great significance. Researchers routinely use quantitative segmentation evaluation metrics such as DSC and HD to compare their results with other proposed algorithms. Although it may be tempting to rely on these measures to draw definitive conclusions on one algorithm’s performance over another, qualitative assessment of results by experts is also necessary to ensure fair judgement and that the clinical demands are met. A few studies incorporated qualitative evaluations to assess the clinical acceptance rate of generated contours [101]; however, this step is not yet widely undertaken for most pelvic cancer segmentation applications.

The generalizability of DL algorithms can be enhanced by use of multi-vendor patient scans for training; however, differences in institutional MR imaging protocols may adversely affect segmentation performance. Contour definition by experts with varying clinical experience (radiologist vs. radiation oncologist) and the source of training data (single- vs. multi-center) are other contributing factors to variabilities in ground-truth ROI delineations which can confound segmentation performance.

The DL-based segmentation publications reviewed in this study proposed improvements in network architectures, image processing techniques, use of multi-parametric input data, loss functions, use of pretrained models (transfer learning) and adversarial training. The fields of DL, particularly computer vision and image segmentation, are still evolving. The industry/application-specific requirements continually encourage innovation and the development of sophisticated networks. The future outlook for pelvic cancer segmentation may include intricate knowledge transfer from pre-trained models on very large datasets or perhaps adaption of key developments from non-medical applications [181] or ones not yet configured for the pelvis [182,183]. The examples of this may include explainable/interpretable AI, domain adaptation and continuous and/or federated learning.

In conclusion, DL in the eyes of clinicians, is still seen as a “black box algorithm” due to its limited interpretability for predicted outcome. Therefore, the clinical adoption of AI-based frameworks is hindered by their lack of interpretability and explainability when generating inaccurate outcomes. Although DL is a powerful and promising tool for many supervised computer-aided applications, it heavily relies on the quality of input data for training. With the absence of standardized and international contouring consensus guidelines to reduce segmentation variabilities, and lack of accessible and annotated public databases, there remains a formidable challenge for true investigation of novel segmentation techniques against existing algorithms. Our review demonstrated the challenges; incentives and public datasets can lead to research contribution from groups from different domains and considerable advancements in technology. Lastly, while embracing the exciting future of DL as a catalyst for a paradigm shift in disease detection, characterization and treatment planning, researchers and clinicians should be aware of the current shortcomings and requirements of automatic pelvic segmentation algorithms in order to push the boundaries of AI in healthcare.

## Figures and Tables

**Table 1:** Summary of previous publications using DL-based automatic segmentation separated by pelvic anatomical regions (Bladder: 6, Cervix: 7, Prostate: 52, Rectum: 9 studies). The DSC and IoU are shown, where reported, with the DSC metrics in bold (for studies with multiple test results, the metrics calculated on public/external databases are presented). For studies that reported neither DSC nor IoU, the metrics used by the authors are included. MRI acquisition modes (2D, 3D) were retrieved based on the information provided in each published article and/or supplementary documents.

Image Modality	Deep Learning Strategy	DL Network Dimension	Number of Patients (Train/Test)	Segmentation Evaluation Metrics	Year	Reference
(MR Acquisition Mode)						
**Bladder Cancer**						
CT	U-Net	2D/3D	81/92	Bladder (IoU: 0.85/0.82)	2019	[88]
CT	CNN + FCN (CRF-RNN)	3D	100/24	Bladder (DSC: 0.92)	2018	[94]
CT	CNN	2D	62 leave-one-out cross validation	Bladder Tumor (area under the ROC curve (AUC): 0.73)	2016	[87]
CT	CNN	2D	81/92	Bladder (IoU: 0.76)	2016	[93]
T_2_W (2D), DW (2D) MRI	AE + modified residual network (BW-Net)	2D	144/25	Bladder Wall (DSC: 0.85)	2020	[96]
T_2_W MRI (3D)	U-Net with progressive dilated convolutions (U-Net Progressive)	2D	40/15	Bladder Tumor (DSC: 0.68), Outer Wall (DSC: 0.83), Inner Wall (DSC: 0.98)	2018	[95]
**Cervical Cancer**						
CT	U-Net with context aggregation blocks (CabUNet)	2D	77/14	Bladder (DSC: 0.90), Bone Marrow (DSC: 0.85), L Fem. Head (DSC: 0.90), R Fem. Head (DSC: 0.90), Rectum (DSC: 0.79), Small Intestine (DSC: 0.83), Spinal Cord (DSC: 0.82)	2020	[97]
CT	Dual path U-Net (DpnUNet)	2.5D	210 five-fold cross validation	CTV (DSC: 0.86), Bladder (DSC: 0.91), Bone Marrow (DSC: 0.85), L Fem. Head (DSC: 0.90), R Fem. Head (DSC: 0.90), Rectum (DSC: 0.82), Bowel Bag (DSC: 0.85), Spinal Cord (DSC: 0.82)	2020	[98]
CT	U-Net	3D	100/25	CTV (DSC: 0.86), Bladder (DSC: 0.88), Rectum (DSC: 0.81), L Fem. Head (DSC: 0.88), R Fem. Head (DSC: 0.88), Small Intestine (DSC: 0.86)	2020	[99]
CT	U-Net with residual connection, dilated convolution and deep supervision (DSD-UNet)	3D	73/18	High-risk CTV (DSC: 0.82, IOU: 0.72), Bladder (DSC: 0.86, IOU: 0.77), Rectum (DSC: 0.82, IOU: 0.71), Small Intestine (DSC: 0.80, IOU: 0.69), Sigmoid (DSC: 0.64, IOU: 0.52)	2020	[100]
CT	V-Net	3D	2464/140 (+30 external test patients)	Primary CTV (UteroCervix) (DSC: 0.85), Nodal CTV (DSC: 0.86), PAN CTV (DSC: 0.76), Bladder (DSC: 0.89), Rectum (DSC: 0.81), Spinal Cord (DSC: 0.90), L Femur (DSC: 0.94), R Femur (DSC: 0.93), L Kidney (DSC: 0.94), R Kidney (DSC: 0.95), Pelvic Bone (DSC: 0.93), Sacrum (DSC: 0.91), L4 Vertebral Body (DSC: 0.91), L5 Vertebral Body (DSC: 0.90)	2020	[101]
MRI (unspecified)	Mask R-CNN	2D	5 (646 images split 9:1 for training and testing)	GTV + Cervix (DSC: 0.84), Uterus (DSC: 0.92), Sigmoid (DSC: 0.89), Bladder (DSC: 0.90), Rectum (DSC: 0.89), Parametrium (DSC: 0.66), Vagina (DSC: 0.71), Mesorectum (DSC: 0.68), Femur (DSC: 0.81)	2019	[102]
DW MRI (2D)	U-Net	2D	144/25	Cervical Tumor (DSC: 0.82)	2019	[17]
**Prostate Cancer**						
CT	U-Net (External commercial software)	2D	328/20	Prostate (DSC: 0.79), Bladder (DSC: 0.97), Rectum (DSC: 0.78), Fem. Head (DSC: 0.91), Seminal Vesicles (DSC: 0.64)	2020	[103]
CT	U-Net	3D	900/30	Prostate (DSC: 0.82), Bladder (DSC: 0.93), Rectum (DSC: 0.84), L Fem. Head (DSC: 0.68), R Fem. Head (DSC: 0.69), Lymph Nodes (DSC: 0.80), Seminal Vesicles (DSC: 0.72)	2020	[104]
CT	High-resolution multi-scale encoder-decoder network (HMEDN)	2D	180/100	Prostate (DSC: 0.88), Bladder (DSC: 0.94), Rectum (DSC: 0.87)	2019	[105]
CT/ Synthetic T_2_W MRI	CT-to-MR synthesis + Deep Attention U-Net (DAUNet)	3D	112/28 five-fold cross validation	Prostate (DSC: 0.87), Bladder (DSC: 0.95), Rectum (DSC: 0.89)	2019	[106]
CT	Modified U-Net	3D	313 five-fold cross validation	Prostate: (DSC: 0.89), Bladder: (DSC: 0.94), Rectum: (DSC: 0.89)	2019	[107]
CT	Deep Neural Network (DNN)	3D	771/140	Prostate (DSC: 0.88)	2019	[108]
CT	Deeply-supervised attention-enabled boosted convolutional neural network (DAB-CNN)	3D	80/20	Prostate (DSC: 0.90), Bladder (DSC: 0.93), Rectum (DSC: 0.83), Penile bulb (DSC: 0.72)	2019	[109]
CT	Distinctive curve guided fully convolutional network (FCN)	2D	313 five-fold cross validation	Prostate (DSC: 0.89), Bladder (DSC: 0.94), Rectum (DSC: 0.89)	2019	[110]
CT	U-Net	2D	60/25	Prostate: (DSC: 0.88), Bladder: DSC: 0.95), Rectum: (DSC: 0.92)	2018	[111]
CT	2D U-Net + 3D U-Net with aggregated residual networks (ResNeXt)	2D/3D	108/28 four-fold cross validation	Prostate (DSC: 0.90), Bladder (DSC: 0.95), Rectum (DSC: 0.84), L Fem. Head (DSC: 0.96), R Fem. Head (DSC: 0.95)	2018	[112]
CT	CNN + multi-atlas fusion	2D	92 five-fold cross validation	Prostate (DSC: 0.86)	2017	[31]
CT	FCN (based on LeNet)	2D	22 two-fold cross validation	Prostate (DSC: 0.89)	2017	[113]
T_2_W MRI (2D)	Adversarial pyramid anisotropic convolutional deep neural network (APA-Net)	3D	110 three-fold cross validation	Whole Prostate Gland (DSC: 0.90)	2020	[114]
T_2_W MRI (2D/3D)	DeeplabV3+	2D	40	Prostate Central Gland (DSC: 0.81), Peripheral Zone (DSC: 0.70)	2020	[115]
T_2_W (2D), DW (2D) MRI	Conditional GAN (cGAN)/Cycle-consistent GAN (Cycle-GAN)	2D	40/50	Whole Prostate Gland (DSC: 0.75)	2020	[116]
T_2_W (2D), DW (2D) MRI	Mask R-CNN	2D	54/16 (+12 external test patients)	Whole Prostate Gland (DSC: 0.86), Prostate Tumor (DSC: 0.56)	2020	[117]
T_2_W MRI (2D)	Boundary-weighted domain adaptive neural network (BOWDA-Net)	3D	40/146	Whole Prostate Gland (DSC: 0.91) Prostate Base (DSC: 0.89) Prostate Apex (DSC: 0.89)	2020	[118]
T_2_W MRI (2D)	Graph convolutional network (GCN)	2D	140 five-fold cross validation	Whole Prostate Gland (DSC: 0.93)	2020	[119]
T_2_W MRI (2D)	Dense U-Net	2D	141/47 four-fold cross validation	Whole Prostate Gland (DSC: 0.92), Central Gland (DSC: 0.89), Peripheral Zone (DSC: 0.78)	2020	[120]
T_2_W MRI (2D)	U-Net/Pix2pix	2D	40 four-fold cross validation	Prostate Central Gland (DSC: 0.86–0.88), Peripheral Zone (DSC: 0.90–0.83)	2020	[121]
T_1_W (3D), T_2_W (unspecified) MRI	Multi-scale DeepMedic	3D	97/53 three-fold cross validation	Bladder (DSC: 0.96), Rectum (DSC: 0.88), L femur (DSC: 0.97), R femur (DSC: 0.97)	2020	[122]
T_2_W MRI (2D)	Cascaded dual attention network (CDA-Net)	3D	40/109	Whole Prostate Gland (DSC: 0.92)	2020	[123]
T_2_W MRI (2D)	Encoder-Decoder structure with dense dilated spatial pyramid pooling (DDSPP)	2D	150	Whole Prostate Gland (DSC: 0.95)	2019	[124]
T_2_W (2D), DW (2D) MRI	Mask R-CNN	2D	36 (split 7:2:1 for training, validation and testing)	Whole Prostate Gland (IoU: 0.84), Prostate Tumor (IoU: 0.40), Central Gland (IoU: 0.78), Peripheral Zone (IoU: 0.51)	2019	[125]
T_2_W (2D), DW (2D) MRI	U-Net	2D	100/125	Whole Prostate Gland (DSC: 0.84), Central Gland (DSC: 0.78), Peripheral Zone (DSC: 0.69)	2019	[126]
T_2_W MRI (2D)	FCN with feature pyramid attention	2D	250/63 (+46 external test patients)	Prostate Transition Zone (DSC: 0.79), Peripheral zone (DSC: 0.74)	2019	[127]
T_2_W MRI (3D)	Spatially-varying stochastic residual adversarial network (STRAINet)	3D	50 five-fold cross validation	Whole Prostate Gland (DSC: 0.91), Bladder (DSC: 0.97), Rectum (DSC: 0.91)	2019	[128]
T_2_W MRI (2D)	U-Net with “combo loss”	3D	700/258	Whole Prostate Gland (DSC: 0.91)	2019	[129]
T_2_W MRI (unspecified)	DeepLabV3+	2D	40/50	CTV (DSC: 0.83), Bladder (DSC: 0.93), Rectum (DSC: 0.82), Penile Bulb (DSC: 0.74), Urethra (DSC: 0.69), Rectal Spacer (DSC: 0.81)	2019	[130]
T_2_W MRI (2D)	V-Net + variational methods	3D	85	Whole Prostate Gland (DSC: 0.64)	2019	[131]
T_2_W MRI (2D)	Propagation Deep Neural Network (P-DNN)	2D	50/30	Whole Prostate Gland: (DSC: 0.84)	2019	[132]
T_2_W (2D), DW (2D) MRI	Cascaded U-Net	2D	76/51	Whole Prostate Gland (DSC: 0.92), Peripheral zone (DSC: 0.79)	2019	[133]
T_2_W MRI (3D)	Multi-view CNN	2D	19 leave-one-out cross validation	Prostate Tumor (DSC: 0.92, IoU: 0.67), Prostate Central Gland (IoU: 0.65), Peripheral Zone (IoU: 0.59)	2019	[134]
T_2_W MRI (2D)	Investigative CNN study (U-Net, V-Net, HighRes3dNet, HolisticNet, Dense V-Net, Adapted U-Net)	3D	173/59	Whole Prostate Gland (DSC: 0.87)	2019	[135]
T_2_W MRI (2D)	Z-Net	2D	45/30	Whole Prostate Gland (DSC: 0.90)	2019	[136]
T_2_W MRI (3D)	FCN	3D	60/10	Whole Prostate Gland (DSC: 0.89), Bladder (DSC: 0.95), Rectum (DSC: 0.88)	2018	[137]
T_2_W MRI (2D)	SegNet	2D	16/5 (+19 external test patients)	Whole Prostate Gland (DSC: 0.75)	2018	[138]
T_2_W MRI (2D)	CNN + Boundary Detection	3D	50 five-fold cross validation	Whole Prostate Gland (DSC: 0.90)	2018	[139]
Dynamic Contrast-Enhanced (DCE) MRI (3D)	U-Net + Long-Short-Term Memory (LSTM)	3D	(15/2) three-fold cross validation	Whole Prostate Gland (DSC: 0.86)	2018	[140]
T_2_W MRI (2D)	FCN	2D	50/30	Whole Prostate Gland (DSC: 0.87)	2018	[141]
T_2_W MRI (2D)	CNN	2D	20	Whole Prostate Gland (DSC: 0.85)	2018	[30]
T_2_W MRI (2D)	CNN (PSNet)	3D	112/28 five-fold cross validation	Whole Prostate Gland (DSC: 0.85)	2018	[29]
T_2_W (2D), DW (2D) MRI	Deep dense multi-path CNN	3D	100/50 (+30 external test patients)	Whole Prostate Gland (DSC: 0.95)	2018	[142]
T_2_W MRI (2D)	U-Net	3D	26	Whole Prostate Gland (DSC: 0.88)	2018	[143]
T_2_W MRI (2D)	Deeply-supervised CNN	2D	77/4	Whole Prostate Gland (DSC: 0.89)	2017	[144]
T_2_W (2D), DW (2D) MRI	Auto-Encoder	2D	21 leave-one-out cross validation	Prostate Tumor (section-based evaluation (SBE): 0.89, sensitivity: 91%, specificity: 88%)	2017	[145]
T_2_W MRI (2D)	Holistically-nested FCN	2D	250 five-fold cross validation	Whole Prostate Gland (DSC: 0.89, IoU: 0.81)	2017	[146]
DW MRI (2D)	Modified U-Net with inception blocks	2D	141 four-fold cross validation	Whole Prostate Gland (DSC: 0.93), Transition Zone (DSC: 0.88)	2017	[147]
T_2_W MRI (2D)	ConvNet with mixed residual connections	3D	50/30	Whole Prostate Gland (DSC: 0.87)	2017	[148]
T_2_W MRI (2D)	Stacked Sparse AE (SSAE) + Sparse patch matching	2D	66 two-fold cross validation	Whole Prostate Gland (DSC: 0.87)	2016	[149]
T_2_W MRI (2D)	V-Net	3D	50/30	Whole Prostate Gland (DSC: 0.87)	2016	[79]
T_2_W MRI (unspecified)	Stacked independent subspace analysis (ISA)	2D	30 leave-one-out cross validation	Whole Prostate Gland (DSC: 0.86)	2013	[150]
**Rectal Cancer**						
CT	DeepLabV3+	2D	98/63	CTV (DSC: 0.88), Bladder (DSC: 0.90), Small Intestine (DSC: 0.76), L Fem. Head (DSC: 0.93), R Fem. Head (DSC: 0.93)	2020	[32]
CT/ T_2_W MRI (2D)	CNN with cascaded atrous convolution (CAC) and spatial pyramid pooling module (SPP)	2D	100/70 five-fold cross validation	Rectal Tumor (DSC: 0.78) CTV (DSC: 0.85)	2018	[151]
CT	Dilated CNN (transfer learning from VGG-16)	2D	218/60	CTV (DSC: 0.87), Bladder (DSC: 0.93), L Fem. Head (DSC: 0.92), R Fem. Head (DSC: 0.92), Intestine (DSC: 0.65), Colon (DSC: 0.62)	2017	[152]
T_2_W (2D), DW (2D) MRI	Mask R-CNN	2D	293/31 (+50 external test patients)	Lymph Nodes (DSC: 0.81)	2020	[153]
T_2_W MRI (2D)	CNN (transfer learning from ResNet50)	2D	461/107	Rectal Tumor (DSC: 0.82)	2019	[154]
T_2_W MRI (3D)	U-Net	2D	93 ten-foldcross validation	Rectal GTV (DSC: 0.74, IoU: 0.60)	2018	[155]
T_2_W MRI (2D)	FCN (transfer learning from VGG-16)	2D	410/102	Rectal Tumor (DSC: 0.84)	2018	[28]
T_2_W MRI (2D)	Hybrid loss FCN (HL-FCN)	3D	64 four-fold cross validation	Rectal Tumor (DSC: 0.72)	2018	[156]
T_2_W (unspecified), DW (2D) MRI	CNN	2D	70/70	Rectal Tumor (DSC: 0.69)	2017	[157]

**Table 2:** Public datasets available for prostate cancer segmentation along with the studies that their results were evaluated on in these databases. T_1_W: T_1_-weighted; T_2_W: T_2_-weighted; DW: Diffusion-weighted; PDW: Proton density-weighted; DCE: Dynamic contrast-enhanced; MRSI: Magnetic resonance spectroscopic imaging.

Dataset	Image Modality (MRI Acquisition Mode)	Number of Patients	Ground-Truth Contours	URL	Studies
PROMISE12 [163]	T_2_W MRI (2D)	80	Whole Prostate Gland	https://promise12.grand-challenge.org/ [Accessed 21 October 2021]	[29,79,114,116,118,119,123,124,128,130,131,132,133,136,141,142,143,147,148]
I2CVB [167]	T_2_W (2D/3D),	40	Whole Prostate Gland, Peripheral Zone, Central Gland, Prostate Tumor	https://i2cvb.github.io/ [Accessed 21 October 2021]	[115,125,134,138,140,168]
	DW (2D),				
	DCE (3D),				
	MRSI (3D) MRI				
BWH [169]	T_1_W (2D/3D),	230	Whole Prostate Gland	https://prostatemrimagedatabase.com/ [Accessed 21 October 2021]	[118,131]
	T_2_W (2D) MRI				
ASPS13 [164]	T_1_W (2D),	156	Whole Prostate Gland, Peripheral Zone	https://wiki.cancerimagingarchive.net/display/Public/NCI-ISBI+2013+Challenge+-+Automated+Segmentation+of+Prostate+Structures [Accessed 21 October 2021]	[29,114,123,124]
	T_2_W (2D),				
	DCE (3D) MRI				
PROSTATEx [165]	T_2_W (2D),	330 (malignant lesions: 76, benign lesions: 245)	Prostate Tumor	https://prostatex.grand-challenge.org/ [Accessed 21 October 2021]	[120,125,127,129]
	DW (2D),				
	PDW (3D),				
	DCE (3D) MRI				

PROMISE12: MICCAI Grand Prostate MR Image Segmentation 2012; I2CVB: Initiative for Collaborative Computer Vision Benchmarking; BWH: The Brigham and Women’s Hospital Database; ASPS13: NCI-ISBI 2013 Challenge for Automatic Segmentation of Prostate Structures; PROSTATEx: SPIE-AAPM-NCI Prostate MR Classification Challenge.
